# Effective discrete-level density matrix model for unipolar quantum optoelectronic devices

**DOI:** 10.1515/nanoph-2024-0766

**Published:** 2025-06-05

**Authors:** Christian Jirauschek

**Affiliations:** TUM School of Computation, Information and Technology, Technical University of Munich (TUM), D-85748 Garching, Germany

**Keywords:** unipolar device, quantum cascade laser, mode-locking, Maxwell-Bloch, linewidth enhancement factor, Bloch gain

## Abstract

Increasingly, unipolar quantum optoelectronic devices such as quantum cascade lasers are employed for the targeted generation of dynamic waveforms in the mid-infrared and terahertz regime. These include for example short-pulse trains, frequency combs and solitons. For the theoretical investigation and targeted development of these devices, suitable semiclassical models such as Maxwell–Bloch type equations have been developed, which employ a two- or multilevel density matrix description for the electron dynamics and a classical propagation equation for the optical resonator field. Unipolar devices typically utilize quantized conduction band states as optical levels. For quantum well and wire structures, the electron states are additionally characterized by a wavevector associated with free motion in the non-confined directions. This degree of freedom can give rise to nonparabolicity effects as well as Bloch gain, both leading to gain asymmetry and linewidth enhancement. However, fully accounting for the wavevector greatly increases the computational cost of the density matrix approach. Here, we introduce an effective discrete-level density matrix model, which includes these effects via correction factors obtained by suitable wavevector averaging. These parameters can be extracted from carrier transport simulations along with other required input data, yielding a self-consistent model. Coupling the effective density matrix description to optical propagation equations results in an effective Maxwell-density matrix approach, which is well-suited for dynamic simulations of quantum optoelectronic devices.

## 1 Introduction

Increasingly, quantum confinement in semiconductor heterostructures is exploited to develop quantum optoelectronic devices with enhanced performance and expanded functionalities. In unipolar devices, the lasing transition occurs between quantized states in the conduction band, and thus the optical properties do not depend on the semiconductor bandgap. This opens up enormous possibilities for custom-tailoring lasing wavelengths, optical nonlinearities and other active region properties by quantum engineering the confined states. Specifically, the quantum cascade laser (QCL) utilizes optical intersubband transitions in the conduction band to access a wide range of mid-infrared (MIR) and terahertz (THz) wavelengths [1], [2]. Here, a periodic multi-quantum well design is used, allowing for the generation of multiple photons by a single injected electron. Also amplifiers [3], [4], modulators [5] and detectors [6], [7] have been realized based on this principle. Generally, unipolar quantum well devices have an enormous potential for long-wavelength optoelectronic applications [8]. Furthermore, also semiconductor quantum wire structures with two-dimensional quantum confinement are attractive candidates for developing intersubband optoelectronics [9].

Recently, dynamic waveform generation with unipolar devices has become a vibrant research field, motivated by a wide range of applications in, e.g., metrology and communications. In particular, mode-locking techniques have been employed for generating short-pulse trains [10], [11] and broadband frequency combs [12], [13], [14], i.e., discrete, equally spaced spectra associated with periodic temporal waveforms. In this context, also harmonic operation in QCLs has attracted considerable interest, where the waveform period is a harmonic of the cavity roundtrip time [15], [16], [17], [18], [19], [20]. Moreover, the formation of dissipative solitons in QCLs has recently caught wide attention [21], [22], [23], [24]. For a systematic design of such waveform-generating nanostructured lasers and improved understanding of their dynamics, accurate and efficient numerical models are essential [25]. More generally, such dynamic modeling approaches are potentially relevant for high-speed systems employing unipolar quantum optoelectronic devices. To account for quantum coherence effects, these approaches are frequently based on a density matrix (DM) formalism describing the electron dynamics in the quantum active region, coupled to Maxwell’s equations capturing the optical field propagation in the cavity. Since often simulations over many hundred or thousand cavity roundtrips are required to reach steady state operation [26], the model is commonly simplified to reduce the numerical load. For example, optoelectronic devices with a waveguide cavity typically feature an invariant transverse field distribution, enabling the use of a one-dimensional optical propagation model which only depends on time *t* and a single spatial coordinate *x* [25]. Furthermore, the dependence on the electron in-plane wavevector **k** is typically ignored in the dynamic DM equations [25], greatly reducing the numerical load in comparison to fully **k** dependent models [27], [28], [29], [30]. This is justified for optical transitions between subbands with nearly parallel dispersion relationships [25], [31], as is often ideally assumed for QCLs [1], but not for interband transitions since the energy dispersions in the conduction and valence bands have opposite curvatures [25], [32]. However, also operation in unipolar quantum well and wire devices can be affected by residual nonparabolicity [33], [34], [35], [36] as well as by Bloch gain [34], [37], [38], [39], [40], both leading to gain asymmetry and linewidth enhancement.

Restricting the description of the quantum active region to two energy levels results in the semi-phenomenological Maxwell-Bloch (MB) equations, which include dissipation in terms of empirical relaxation rates [25]. Various strategies have been employed to derive effective MB equations for bipolar semiconductor lasers and amplifiers from microscopic models by adequate **k** averaging over the electron and hole distributions [32], [41], [42]. These models include a linewidth enhancement factor (LEF) to describe nonparabolicity effects. For unipolar devices, the so-called effective semiconductor MB equations (ESMBEs) have been derived by combining the MB equations with a phenomenological expression for an asymmetric material susceptibility [43], [44], and employed for studying the dynamic QCL operation in both ring and Fabry–Perot configurations [35], [43], [44], [45], [46]. Also the Bloch gain has been implemented in the MB framework [39]. On the other hand, fully quantitative modeling of quantum-engineered optoelectronic devices requires explicit consideration of all relevant mechanisms and quantized energy levels. This can be achieved in the framework of an advanced Maxwell-DM model, featuring a multilevel DM and a generalized system Hamiltonian, which generally includes tunneling in addition to light–matter interaction [25], [47]. Dissipation is here described using the Lindblad formalism [25], [48]. The Lindblad-type relaxation terms and Hamiltonian matrix elements can be extracted from carrier transport simulations or microscopic descriptions, resulting in a self-consistent device model [26], [31]. This approach has been employed for quantitative simulations of various advanced THz and MIR QCL devices in Fabry–Perot and ring configurations, yielding excellent agreement with experiment and providing insights into device operation. Examples include the modeling of soliton generation [22], short-pulse mode-locked operation [11], [47], and fundamental [26], [31], harmonic [20], [49] as well as difference-frequency comb [50] generation. The multilevel DM naturally includes gain asymmetry due to multiple optical transitions, which can have a significant influence on the optical dynamics [17]. However, contributions of nonparabolicity and Bloch gain have to date not been considered in Maxwell-DM approaches beyond the two-level approximation. In the present work, these effects are systematically incorporated by suitable **k** averaging of the microscopic DM equations. The resulting correction factors, such as effective transition frequencies and LEF-related quantities, are in our approach not treated as fitting parameters, but can be extracted from carrier transport simulations together with the other required parameters. Thus, the resulting effective Maxwell-DM equations preserve the self-consistent nature of the simulation model.

## 2 Microscopic model

**Figure 1:**
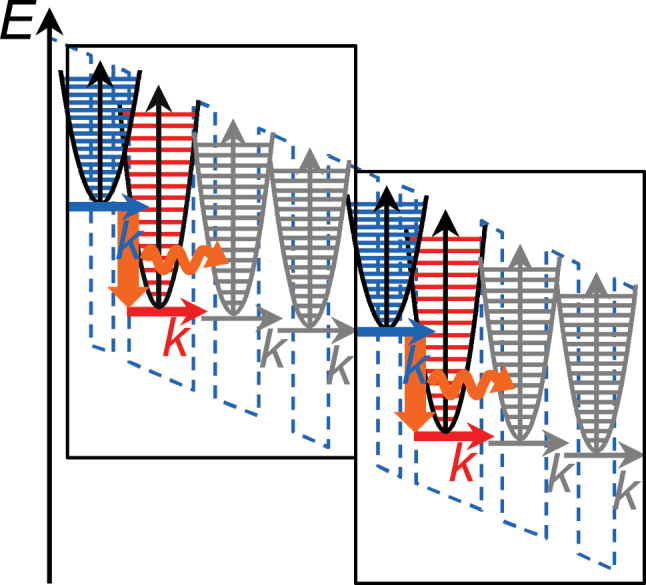
Schematic representation of level schemes for unipolar devices. Additionally, parabolic electron dispersion relations as given in Eq. (1) are illustratively sketched. The upper and lower levels of the optical transition are indicated by blue and red colors, respectively. For QCLs, a periodic repetition of identical stacks (marked by rectangles) is used.

For interband transitions, the derivation of effective two-level models by suitable wavevector summation has been addressed in previous work [32], [41], [42]. Here, we focus on unipolar devices. As illustrated in Figure 1, these utilize optical transitions between quantized energy levels *n*, each consisting of a quasi-continuum of states $\left|n,k\right\rangle$ with eigenenergies *E*_*n*,**k**_. The associated transition frequencies are given by $\omega_{mn,k}=\left(E_{m,k}-E_{n,k}\right)/\hbar$ , with the reduced Planck constant *ℏ*. Specifically for quantum well structures, quantum confinement in growth direction *z* results in the formation of quantized states *n*, and the free in-plane carrier motion is described by the two-dimensional in-plane wavevector **k**. Nonparabolicity can be included by allowing for an energy dependent effective mass when solving the Schrödinger equation. This yields for each subband *n* the corresponding wavefunction $\psi_{n}\left(z\right)$ and the electron dispersion relation

(1) $$E_{n,k}=E_{n}+\hbar^{2}{\left|k\right|}^{2}/\left(2m_{n}^{*}\right),$$

with the subband effective mass $m_{n}^{*}$ and *E*_*n*_ = *E*_*n*,**k**=**0**_ [51], [52]. Here, we assume decoupling between the in-plane motion and the confinement direction implying **k** independent wavefunctions $\psi_{n}\left(z\right)$ , which is a good approximation for not too narrow finite quantum wells [53]. Within this model, the effect of nonparabolicity is accounted for by the different value of $m_{n}^{*}$ for each subband. We note however that the treatment of nonparabolicity in our effective DM equations derived in Section 3.2 is not restricted to dispersion relations of the form Eq. (1).

The DM elements are given by $\left(m,k\right|\hat{\rho }\left|n,k\right\rangle =\rho_{mn,k}$ , and reduction to an effective discrete-level description using DM elements of the form *ρ*_*mn*_ requires suitable **k** averaging. Also semiconductor quantum wire structures with two-dimensional quantum confinement are attractive candidates for developing intersubband devices [9]. They can be described analogously; in this case, the free carrier motion in the remaining direction is characterized by a one-dimensional wavevector [25]. Besides, our approach is not restricted to the parabolic dispersion relations given in Eq. (1).

The diagonal DM elements *ρ*_*nn*,**k**_ = *ρ*_*n*,**k**_ can be written as

(2) $$\rho_{n,k}=\rho_{n}f_{n,k}/\underset{k}{\sum }f_{n,k}=Sf_{n,k},$$

where the distribution function *f*_*n*,**k**_ gives the electron occupation probability of a state $\left|n,k\right\rangle$ . The scaling factor *S* may be chosen such that *ρ*_*n*_ = *S*∑_**k**_ *f*_*n*,**k**_ corresponds, e.g., to the carrier number density in level *n*, or that the normalization condition ∑*ρ*_*n*_ = 1 is fulfilled. The off-diagonal elements *ρ*_*ij*,**k**_ contain the coherence between states $\left|i,k\right\rangle$ and $\left|j,k\right\rangle$ . The DM evolution equation is given by

(3) $$\partial_{t}\hat{\rho }=-\frac{i}{\hbar }\left[\hat{H},\hat{\rho }\right]+{\left[\partial_{t}\hat{\rho }\right]}_{\text{col}},$$

with the collision term ${\left[\partial_{t}\hat{\rho }\right]}_{\text{col}}$ . DM elements between different wavevectors need not be considered due to the **k** conservation of optical transitions. This also applies for first-order tunneling processes, which can straightforwardly be included in the Hamiltonian by employing adequately localized basis states [54], [55]. For an orthogonal basis set, we obtain from Eq. (3)

(4) $$\partial_{t}\rho_{mn,k}=\frac{i}{\hbar }\underset{i}{\sum }\left(\rho_{mi,k}H_{in,k}-H_{mi,k}\rho_{in,k}\right)+{\left[\partial_{t}\rho_{mn,k}\right]}_{\text{col}}.$$

The Hamiltonian in Eq. (4) can be represented as $\hat{H}={\hat{H}}_{0}+{\hat{H}}_{\text{I}}$ , where ${\hat{H}}_{0}$ is the Hamiltonian of the unperturbed system, with *H*_0,*nn*,**k**_ = *E*_*n*,**k**_. Off-diagonal elements can, e.g., arise from the inclusion of resonant tunneling. For example, in QCL designs electron transport across thick injection or extraction barriers is mediated by tunneling between closely aligned states. Using a localized basis, such as tight-binding states $\left|m,k\right\rangle$ and $\left|n,k\right\rangle$ located at the left and right of the barrier [55], [56] or EZ states [57], the corresponding DM elements can for small $\left|\omega_{mn,k}\right|$ be written as *H*_0,*mn*_ = *H*_0,*nm*_ = *ℏ*Ω_*mn*_. Assuming **k** independent wavefunctions as discussed above, also the coupling energy *ℏ*Ω_*mn*_ = *ℏ*Ω_*nm*_ does not depend on **k** [55], [56]. For an optical transition between two states $\left|m,k\right\rangle$ and $\left|n,k\right\rangle$ , light–matter interaction can in dipole approximation be described by the corresponding matrix elements of the interaction Hamiltonian, *H*_I,*mn*_ = *H*_I,*nm*_ = −*Ed*_*mn*_, with the optical field $E\left(t\right)$ . Here, *d*_*mn*_ = *d*_*nm*_ represents the dipole matrix element, which is again **k** independent under above assumptions. The model for the collision term in Eq. (4) used here is discussed in Appendix A. We note that within above framework, second-order effects connecting states with different wavevectors, such as Bloch gain and second-order tunneling, are not yet included. Various approaches have been discussed in literature to consider these contributions in effective discrete-level DM models [39], [58], [59]. In Section 3.2.1, we give a detailed discussion on the implementation of Bloch gain.

In the following, we restrict our discussions to a field *E* with moderate bandwidth and in close resonance with the optical transition(s), i.e., $\omega_{\text{c}}\approx \left|\omega_{mn,k}\right|$ where *ω*_c_ denotes the optical carrier frequency. Furthermore assuming non-excessive field strengths as is typically justified in optoelectronics, the widely used rotating wave approximation (RWA) can be invoked to increase the numerical efficiency of the model [25]. Here, the fast oscillations of *E* and the related DM elements *ρ*_*mn*,**k**_ around *ω*_c_ can be removed by representing these quantities in terms of the slowly varying envelope functions *ɛ*_*mn*_ and *η*_*mn*,**k**_,

(5a) $$\rho_{mn,k}=\eta_{mn,k}\exp\left[-i\omega_{\text{c}}\mathrm{sgn}\left(\omega_{mn,k}\right)t\right],$$

(5b) $$d_{mn}E/\hbar =\left[\varepsilon_{mn}\exp\left(-i\omega_{\text{c}}t\right)+\varepsilon_{mn}^{*}\exp\left(i\omega_{\text{c}}t\right)\right]/2.$$

More specifically, the field envelope *ɛ*_*mn*_ = *ɛ*_*nm*_ is here expressed in terms of the corresponding (instantaneous) Rabi frequency. The asterisk denotes the complex conjugate, and sgn represents the sign function. The evolution equations for the DM elements in RWA are then obtained in the usual manner by substituting Eqs. (5a) and (5b) into (4) and discarding the rapidly oscillating terms (see Appendix B). Since a coarser spatiotemporal grid can be used to resolve the dynamics of the envelope functions, the computational load gets significantly reduced as compared to full-wave simulations.

## 3 Effective discrete-level model

For the DM-based dynamic modeling of semiconductor lasers and other optoelectronic devices, typically a two- or multilevel model featuring discrete energy levels is used, where the wavevector dependence of the states is not explicitly taken into account [25]. Besides the considerable decrease of numerical complexity as compared to fully microscopic models [25], discrete-level approaches facilitate the development of compact and intuitive descriptions of the laser dynamics [45], [60], [61], [62]. However, the wavevector dependence may leave a direct imprint on the DM dynamics beyond microscopic interactions, e.g., in form of an asymmetric susceptibility and the closely related linewidth enhancement resulting from the nonparabolicity effect or Bloch gain [32], [35], [39], [43]. Thus, rather than simply ignoring the wavevector dependence of the microscopic states, a systematic removal of this quantity from the model by adequate **k** summation is more appropriate.

**Figure 2:**
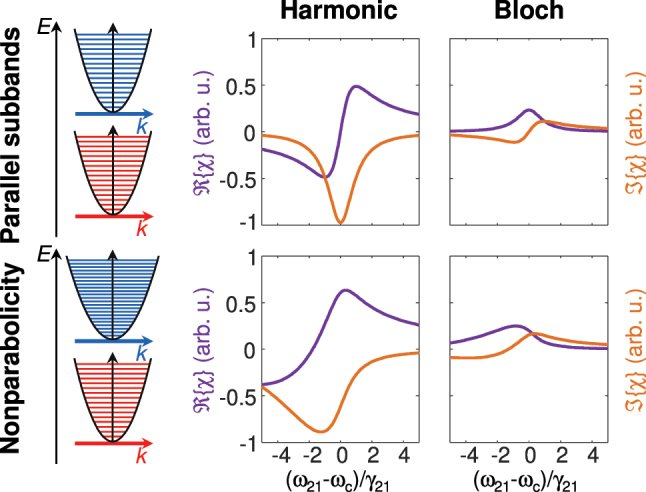
Schematic representation of harmonic and Bloch contribution to the susceptibility *χ* for parabolic subbands and for nonparabolicity. Here, *ω*_21_ and *γ*_21_ are the resonance frequency at **k** = **0** and the dephasing rate.

The transition from the microscopic, **k**-resolved description to an effective model is achieved by defining effective DM elements obtained via **k** summation,

(6) $$\begin{array}{ll} \rho_{mn} & =\underset{k}{\sum }\rho_{mn,k},\\ \eta_{mn} & =\underset{k}{\sum }\eta_{mn,k}, \end{array}$$

where the diagonal DM elements, *ρ*_*nn*_ ≕ *ρ*_*n*_, are related to the total population of level *n*, and the elements *η*_*mn*_ to the polarization of the optical transition *m* → *n*. For a stationary optical field with frequency *ω*, *η*_*mn*_ is directly proportional to *χɛ*_*mn*_ with the complex susceptibility $\chi \left(\omega \right)$ . In Figure 2, *χ* is schematically illustrated for the case of parallel subbands and for nonparabolicity, resulting from different effective masses of the upper and lower subband. Here, population inversion is assumed. For the complex field convention introduced in Eq. (5b), $I\left\{\chi \right\}$ is proportional to the loss coefficient. For parallel subbands, both the harmonic gain and the real part of the Bloch susceptibility assume the typical Lorentzian shape. An asymmetric susceptibility is obtained for nonparabolicity, or also for parallel subbands if both the harmonic and Bloch contributions are present.

### 3.1 Populations

Since the equations for the level populations (see Eq. (B.1) in Appendix B) do not contain products of **k** dependent quantities, **k** summation can be directly performed, resulting in

(7) $$\begin{array}{ll} \partial_{t}\rho_{n} & =i\underset{i\neq n}{\sum }\left(\Omega_{in}\rho_{ni}-\Omega_{ni}\rho_{in}\right)\\ & \;+\underset{\begin{array}{c} i\\ \omega_{ni}>0 \end{array}}{\sum }I\left\{\varepsilon_{ni}^{*}\eta_{ni}\right\}+\underset{\begin{array}{c} i\\ \omega_{ni}<0 \end{array}}{\sum }I\left\{\varepsilon_{ni}\eta_{ni}\right\}\\ & \;+\underset{i\neq n}{\sum }r_{in}\rho_{i}-\rho_{n}\underset{i\neq n}{\sum }r_{ni}. \end{array}$$

The collision term describing intersubband scattering in Eq. (B.1) is here modeled using Eq. (A.1b), and **k** averaging yields

(8) $${\left[\partial_{t}\rho_{n}\right]}_{\text{col}}=\underset{i\neq n}{\sum }r_{in}\rho_{i}-\rho_{n}\underset{i\neq n}{\sum }r_{ni}.$$

The effective rates *r*_*mn*_ are given by

(9) $$r_{mn}=\underset{k,q}{\sum }W_{mk,nq}f_{m,k}^{\text{o}}\left(1-f_{n,q}^{\text{o}}\right)/\underset{k}{\sum }f_{m,k}^{\text{o}},$$

where *W*_*m* **k**,*n* **q**_ denotes the microscopic scattering rate from a state $\left|m,k\right\rangle$ to $\left|n,q\right\rangle$ . The resulting *r*_*mn*_ are already corrected for Pauli blocking, which can however often be neglected in QCLs due to the relatively low doping levels [52]. The rates for various relevant intersubband scattering mechanisms in quantum well and wire structures, such as electron–electron, electron–phonon and electron-impurity interactions, have been discussed in literature [52], [63], [64]. We note that the inclusion of carrier–carrier scattering yields rates which are themselves dependent on the carrier distribution [52], [63]. For simplicity, constant rates *r*_*mn*_ have been assumed in Eq. (9) by replacing the carrier distributions *f*_*i*,**k**_ with their values $f_{i,k}^{\text{o}}$ at the operating point. For self-consistent modeling, these can be extracted from fully **k** dependent stationary carrier transport simulations, implying that the temporal modulation of the carrier populations around their steady-state values is not excessive [47], [56].

### 3.2 Two-level coherence

Let us assume an intersubband optical transition with a single upper and lower level *u* and *ℓ*, which are coupled to other levels only by incoherent scattering transitions. In Figure 1, this corresponds to the case where the optical levels are not coherently coupled to further states. Such a transition can be described by an open two-level quantum system. Indeed, available models for the QCL dynamics including gain asymmetry and linewidth enhancement are commonly based on a two-level quantum system approach [35], [39], [43]. Under above assumptions, Eq. (7) simplifies to

(10) $$\begin{array}{ll} \partial_{t}\rho_{n} & =\mathrm{sgn}\left(\omega_{nm}\right)I\left\{\varepsilon_{u\ell }^{*}\eta_{u\ell }\right\}\\ & \;+\underset{i\neq n}{\sum }r_{in}\rho_{i}-\rho_{n}\underset{i\neq n}{\sum }r_{ni}, \end{array}$$

with *n* = *u*, *m* = *ℓ* and *m* = *u*, *n* = *ℓ*, respectively. For the two-level case, Eq. (B.3) simplifies to

(11) $$\begin{array}{ll} \partial_{t}\eta_{u\ell ,k} & =-s_{u\ell ,k}\eta_{u\ell ,k}\\ & \;+\frac{i}{2}\varepsilon_{u\ell }\left(\rho_{\ell ,k}-\rho_{u,k}\right). \end{array}$$

A straightforward **k** summation is impeded by the term *s*_*uℓ*,**k**_ *η*_*uℓ*,**k**_. A naive ansatz, where ∑_**k**_ *s*_*uℓ*,**k**_ *η*_*uℓ*,**k**_ is approximated by a term *s*_*uℓ*,eff_ *η*_*uℓ*_ with some complex-valued parameter $s_{u\ell ,e\mathrm{ff}}=\gamma_{u\ell ,e\mathrm{ff}}+i\left(\omega_{u\ell ,e\mathrm{ff}}-\omega_{\text{c}}\right)$ , is not productive since this just leads to a modified resonance frequency and dephasing of the transition, but not to an asymmetric lineshape. Instead, we apply the approach by Yao et al. [32], originally developed to describe the nonparabolicity of optical transitions between the conduction and valence bands. Here, both sides of Eq. (11) are divided by *s*_*uℓ*,**k**_, and subsequently, the **k** summation is performed. This yields after multiplication with Γ_*uℓ*_

(12) $$\partial_{t}\eta_{u\ell }=-\Gamma_{u\ell }\eta_{u\ell }+\frac{i}{2}\varepsilon_{u\ell }\left(c_{u\ell ,\ell }^{\text{np}}\rho_{\ell }-c_{u\ell ,u}^{\text{np}}\rho_{u}\right),$$

where the nonparabolicity parameter is given by $c_{u\ell ,i}^{\text{np}}=\Gamma_{u\ell }\tau_{u\ell ,i}^{\text{np}}$ , and

(13a) $$\tau_{u\ell ,i}^{\text{np}}=\underset{k}{\sum }s_{u\ell ,k}^{-1}\rho_{i,k}/\rho_{i},$$

(13b) $$\Gamma_{u\ell }=\eta_{u\ell }/\underset{k}{\sum }s_{u\ell ,k}^{-1}\eta_{u\ell ,k}.$$

Rather than using $c_{u\ell ,i}^{\text{np}}$ and Γ_*uℓ*_ in Eq. (12) as fitting parameters to experimental data, we derive them from fully **k** dependent stationary carrier transport modeling at the operating point of the device, similarly as for the rates in Eq. (9). Here, we use the corresponding results for the carrier distributions $f_{i,k}^{\text{o}}$ and populations $\rho_{i}^{\text{o}}$ along with the obtained dephasing rates to evaluate Eq. (13). While *τ*_*uℓ*,*i*_ can be straightforwardly calculated from Eq. (13a), Eq. (13b) requires computing the stationary value of *η*_*uℓ*,**k**_ and *η*_*uℓ*_ at the operating point by setting ∂_*t*_ = 0 in Eqs. (11) and (12), respectively. This yields with Eq. (2)

(14a) $$\eta_{u\ell ,k}^{\text{o}}=\frac{i}{2}S\varepsilon_{u\ell }s_{u\ell ,k}^{-1}\left(f_{\ell ,k}^{\text{o}}-f_{u,k}^{\text{o}}\right),$$

(14b) $$\eta_{u\ell }^{\text{o}}=\frac{i}{2}\varepsilon_{u\ell }\Gamma_{u\ell }^{-1}\left(c_{u\ell ,\ell }^{\text{np}}\rho_{\ell }^{\text{o}}-c_{u\ell ,u}^{\text{np}}\rho_{u}^{\text{o}}\right).$$

Thus, we obtain from Eq. (13) the nonparabolicity parameters

(15a) $$\tau_{u\ell ,i}^{\text{np}}=\underset{k}{\sum }s_{u\ell ,k}^{-1}f_{i,k}^{\text{o}}/\underset{k}{\sum }f_{i,k}^{\text{o}},$$

(15b) $$\Gamma_{u\ell }=\frac{\underset{k}{\sum }s_{u\ell ,k}^{-1}\left(f_{u,k}^{\text{o}}-f_{\ell ,k}^{\text{o}}\right)}{\underset{k}{\sum }s_{u\ell ,k}^{-2}\left(f_{u,k}^{\text{o}}-f_{\ell ,k}^{\text{o}}\right)}.$$

For modeling the combined optical and electronic device dynamics in a self-consistent manner, the DM model is coupled to optical propagation equations for the resonator field, where also spatial hole burning (SHB) arising from standing-wave patterns must be considered (see Appendix C).

#### 3.2.1 Bloch gain

In the following, we assume a quantum well structure with in-plane isotropy, which is generally justified for direct bandgap semiconductors as used for QCLs. Thus, the electron energies, distribution functions, dephasing rates etc. just depend on the wavevector magnitude $k=\left|k\right|$ . We furthermore restrict ourselves to a parabolic dispersion relation for each subband, described in Eq. (1). The inclusion of Bloch gain into Eq. (14a) then yields the total DM element [37], [39]

(16) $$\eta_{u\ell ,k}^{t}=\eta_{u\ell ,k}^{\text{o}}+\frac{1}{2}S\varepsilon_{u\ell }\left(g_{u\ell ,uk}-g_{u\ell ,\ell k}\right),$$

with

(17) $$g_{u\ell ,ik}=\frac{\gamma_{i,k}\left(f_{i,k_{i}}^{\text{o}}-f_{i,k}^{\text{o}}\right)H\left(k_{i}^{2}\right)}{\delta_{u\ell ,k}s_{u\ell ,k}},$$

and

$$\begin{array}{ll} k_{u}^{2} & =\left(m_{u}^{*}/m_{\ell }^{*}\right)k^{2}-2m_{u}^{*}\hbar^{-1}\delta_{u\ell ,k=0},\\ k_{\ell }^{2} & =\left(m_{\ell }^{*}/m_{u}^{*}\right)k^{2}+2m_{\ell }^{*}\hbar^{-1}\delta_{u\ell ,k=0}. \end{array}$$

*H* denotes the Heaviside step function, *δ*_*uℓ*,*k*_ and *s*_*uℓ*,*k*_ are defined in Eq. (B.4), and *γ*_*i*,*k*_ is the broadening of state $\left|i,k\right\rangle$ , with *γ*_*uℓ*,*k*_ = *γ*_*u*,*k*_ + *γ*_*ℓ*,*k*_. We make the ansatz

(18) $$\begin{array}{ll} \partial_{t}\eta_{u\ell } & =-\Gamma_{u\ell }\eta_{u\ell }+\frac{i}{2}\varepsilon_{u\ell }\left[c_{u\ell ,\ell }\rho_{\ell }-c_{u\ell ,u}\rho_{u}\right],\\ c_{u\ell ,i} & =c_{u\ell ,i}^{\text{np}}+c_{u\ell ,i}^{\text{b}}=\Gamma_{u\ell }\left(\tau_{u\ell ,i}^{\text{np}}+\tau_{u\ell ,i}^{\text{b}}\right), \end{array}$$

where the parameter $c_{u\ell ,i}^{\text{b}}=\Gamma_{u\ell }\tau_{u\ell ,i}^{\text{b}}$ represents the Bloch gain, while $c_{u\ell ,i}^{\text{np}}=\Gamma_{u\ell }\tau_{u\ell ,i}^{\text{np}}$ is the nonparabolicity parameter obtained from Eq. (15). Setting ∂_*t*_ = 0 in Eq. (18) yields the stationary solution

(19) $$\eta_{u\ell }^{t}=\eta_{u\ell }^{\text{o}}-\frac{i}{2}\varepsilon_{u\ell }\Gamma_{u\ell }^{-1}\left(c_{u\ell ,u}^{\text{b}}\rho_{u}^{\text{o}}-c_{u\ell ,\ell }^{\text{b}}\rho_{\ell }^{\text{o}}\right),$$

where the second contribution contains the Bloch gain. We thus obtain with Eqs. (16) and (19)

(20) $$\tau_{u\ell ,i}^{\text{b}}=i\frac{\underset{k}{\sum }g_{u\ell ,ik}}{\underset{k}{\sum }f_{i,k}^{\text{o}}}.$$

#### 3.2.2 Interpretation

The meaning of the physical parameters in Eq. (18), and correspondingly in Eq. (12), can be understood from calculating *η*_*uℓ*_, which is closely related to the complex susceptibility *χ*, as a function of the detuning frequency Δ = *ω* − *ω*_c_. To this end, we insert a frequency-detuned field $\varepsilon_{u\ell }\to \varepsilon_{u\ell }\left(\Delta \right)\exp\left(-i\Delta t\right)$ and the corresponding DM element $\eta_{u\ell }\to \eta_{u\ell }\left(\Delta \right)\;\exp\left(-i\Delta t\right)$ into Eq. (18). After cancelling $\exp\left(-i\Delta t\right)$ from both sides, we obtain the stationary solution (∂_*t*_ = 0)

(21) $$\eta_{u\ell }\left(\Delta \right)=\frac{1}{2}\varepsilon_{u\ell }\left(\Delta \right)\frac{c_{u\ell ,u}\rho_{u}-c_{u\ell ,\ell }\rho_{\ell }}{\Delta +i\Gamma_{u\ell }}.$$

By analogy with Eq. (B.4) we can express Γ_*uℓ*_ as

(22) $$\Gamma_{u\ell }=\gamma_{u\ell }^{\text{e}}+i\left(\omega_{u\ell }^{\text{e}}-\omega_{\text{c}}\right),$$

i.e., $\gamma_{u\ell }^{\text{e}}$ and $\omega_{u\ell }^{\text{e}}\;$ are the effective dephasing rate and resonance frequency in the effective discrete-level model. For simulations featuring a single optical transition, the optical carrier frequency can be chosen as $\omega_{\text{c}}=\omega_{u\ell }^{\text{e}}$ , such that Eq. (22) simplifies to $\Gamma_{u\ell }=\gamma_{u\ell }^{\text{e}}$ . This is not possible in devices featuring heterogeneous active regions, or multiple sections with different transition frequencies. To recover the usual dependence of Eqs. (18) and (21) on the population inversion *ρ*_*u*_ − *ρ*_*ℓ*_, as found in the conventional MB equations [25], we can write the population dependent term appearing in Eqs. (21) and (18) as

(23) $$c_{u\ell ,u}\rho_{u}-c_{u\ell ,\ell }\rho_{\ell }=c_{u\ell }\left(\rho_{u}-\rho_{\ell }\right).$$

Evaluating Eq. (23) at the operating point yields

(24) $$c_{u\ell }=\frac{c_{u\ell ,u}\rho_{u}^{\text{o}}-c_{u\ell ,\ell }\rho_{\ell }^{\text{o}}}{\rho_{u}^{\text{o}}-\rho_{\ell }^{\text{o}}}.$$

Away from the operating point, the right-hand side of Eq. (23) with *c*_*uℓ*_ given in Eq. (24) is a good approximation if *c*_*uℓ*,*u*_ ≈ *c*_*uℓ*,*ℓ*_, or if the population in one of the two levels is negligible. For example, *ρ*_*ℓ*_ ≈ 0 is assumed in the ESMBEs [43], [44]. The general form of the population dependence given by the left-hand side of Eq. (23) can be decomposed into two terms $\propto \left(\rho_{u}-\rho_{\ell }\right)$ and $\propto \left(\rho_{u}+\rho_{\ell }\right)$ , respectively. In the context of the Bloch gain, it has been noted that the contribution $\propto \left(\rho_{u}+\rho_{\ell }\right)$ can lead to residual gain even without population inversion [39].

From Eqs. (21) and (23), we find that $I\left\{c_{u\ell }/\left(\Delta +i\Gamma_{u\ell }\right)\right\}$ at the operating point has an extremum for the detuning frequency

(25) $$\Delta_{p}=\left(\omega_{u\ell }^{\text{e}}-\omega_{\text{c}}\right)+\gamma_{u\ell }^{\text{e}}\left(x-\sqrt{x^{2}+1}\right)$$

with $x=R\left\{c_{u\ell }\right\}/I\left\{c_{u\ell }\right\}$ , corresponding to the gain (or absorption) peak.

For computing the linewidth enhancement factor, we must consider that intensity-induced changes *δρ*_*u*_ and *δρ*_*ℓ*_ of the upper and lower laser level populations at a given working point are generally related via *δρ*_*ℓ*_ = −*ζδρ*_*u*_, where the factor *ζ* can be extracted from the scattering, optical and tunneling rates in the system [65]. Specifically, *ζ* = 0 for ideal depopulation of the lower laser level, and *ζ* = 1 for a closed two-level model where *ρ*_*u*_ + *ρ*_*ℓ*_ is preserved. Using that the susceptibility *χ* is proportional to *η*_*uℓ*_/*ɛ*_*uℓ*_ and taking the complex field convention introduced in Eq. (5b), we obtain with Eq. (21) the frequency dependent linewidth enhancement factor

(26) $$\begin{array}{ll} \alpha & =\frac{\partial_{\rho_{u}}R\left\{\chi \right\}}{\partial_{\rho_{u}}I\left\{\chi \right\}}\\ & =\frac{R\left\{\left(c_{u\ell ,u}+\zeta c_{u\ell ,\ell }\right)\left(\omega -\omega_{u\ell }^{\text{e}}-i\gamma_{u\ell }^{\text{e}}\right)\right\}}{I\left\{\left(c_{u\ell ,u}+\zeta c_{u\ell ,\ell }\right)\left(\omega -\omega_{u\ell }^{\text{e}}-i\gamma_{u\ell }^{\text{e}}\right)\right\}}. \end{array}$$

#### 3.2.3 Analytical evaluation of parameters

Under certain assumptions, the parameters $\tau_{u\ell ,i}^{\text{np}}$ , Γ_*uℓ*_ and $\tau_{u\ell ,i}^{\text{b}}$ given in Eqs. (15) and (20) can be analytically computed. Similarly as in Section 3.2.1, we restrict the discussion to a quantum well structure with a parabolic dispersion relation of the form Eq. (1) for each subband. Nonparabolicity related to different effective masses of the laser levels then yields with Eqs. (1) and (B.4)

(27) $$s_{u\ell }=s_{u\ell ,0}+s_{u\ell }^{\prime }w=\gamma_{u\ell }+i\delta_{u\ell ,0}+i\delta_{u\ell }^{\prime }w,$$

where *δ*_*uℓ*,0_ = *ω*_*uℓ*,**k=0**_ − *ω*_c_ and $\delta_{u\ell }^{\prime }=\hbar^{-1}m_{\text{e}}\left[{\left(m_{u}^{*}\right)}^{-1}-{\left(m_{\ell }^{*}\right)}^{-1}\right]$ . Here, we have introduced an energy variable

(28) $$w=\frac{\hbar^{2}{\left|k\right|}^{2}}{2m_{\text{e}}},$$

defined such that the in-plane kinetic energy in a subband *i* is given by $wm_{\text{e}}/m_{i}^{*}$ where *m*_e_ is the electron mass. If we can also describe the dephasing part of Eq. (27) by a linear energy dependence, $\gamma_{u\ell }=\gamma_{u\ell ,0}+\gamma_{u\ell }^{\prime }w\;$ with $\gamma_{u\ell ,0}=\gamma_{u\ell }\left(w=0\right)$ and $\gamma_{u\ell }^{\prime }=\partial_{w}\gamma_{u\ell }\left(w=0\right)$ , we have *s*_*uℓ*,0_ = *γ*_*uℓ*,0_ + i*δ*_*uℓ*,0_ and $s_{u\ell }^{\prime }=\gamma_{u\ell }^{\prime }+i\delta_{u\ell }^{\prime }$ in Eq. (27). Analogously, we can use $\gamma_{i}=\gamma_{i,0}+\gamma_{i}^{\prime }w$ in Eq. (17). However, the linear approximation does not always provide a good fit for the dephasing, in which case it is better to describe *γ*_*uℓ*_ and *γ*_*i*_ by a constant, suitably averaged value [56]. Furthermore, we assume that the kinetic electron distributions are thermalized and can thus for each subband *i* be characterized by an electron temperature *T*_*i*_ [52]. For moderate doping levels, as is often the case in QCLs, *f*_*i*_ in Eq. (2) is then approximately given by a Maxwell-Boltzmann distribution [52]. For analytical evaluation, we express Eq. (2) as

$$\rho_{i}\left(w\right)=m_{\text{e}}{\left(m_{i}^{*}k_{\text{B}}T_{i}\right)}^{-1}\rho_{i}\exp\left[-wm_{\text{e}}/\left(m_{i}^{*}k_{\text{B}}T_{i}\right)\right]$$

with the Boltzmann constant *k*_B_, and replace the sums $\sum_{k}\ldots f_{i,k}^{\text{o}}$ in Eqs. (15) and (20) by integrals over *w*. Defining

$$\begin{array}{ll} I\left(a,b\right) & =\overset{\infty }{\underset{0}{\int }}\frac{\exp\left(-x\right)}{a+bx}dx=b^{-1}E_{1}\left(a/b\right)\exp\left(a/b\right),\\ J\left(a,b\right) & =\overset{\infty }{\underset{0}{\int }}\frac{\exp\left(-x\right)}{{\left(a+bx\right)}^{2}}dx=b^{-1}\left[a^{-1}-I\left(a,b\right)\right] \end{array}$$

with the exponential integral $E_{1}\left(x\right)=\int_{1}^{\infty }t^{-1}\exp\left(-xt\right)dt$ , we can express Eq. (15) as

(29a) $$\tau_{u\ell ,i}^{\text{np}}=I\left(s_{u\ell ,0},s_{u\ell }^{\prime }w_{i}\right),$$

(29b) $$\Gamma_{u\ell }=\frac{\rho_{u}^{\text{o}}\tau_{u\ell ,u}^{\text{np}}-\rho_{\ell }^{\text{o}}\tau_{u\ell ,\ell }^{\text{np}}}{\rho_{u}^{\text{o}}J\left(s_{u\ell ,0},s_{u\ell }^{\prime }w_{u}\right)-\rho_{\ell }^{\text{o}}J\left(s_{u\ell ,0},s_{u\ell }^{\prime }w_{\ell }\right)},$$

where $w_{i}=k_{\text{B}}T_{i}m_{i}^{*}/m_{\text{e}}$ . Furthermore defining the function $G\left(a,b,c,d,\mu ,x_{0}\right)$ as

(30) $$\begin{array}{ll} G & =\overset{\infty }{\underset{x_{0}}{\int }}\frac{\left(c+dx\right)\exp\left(-\mu x\right)}{I\left\{a+bx\right\}\left(a+bx\right)}dx\\ & =\frac{cb-da}{\left(a_{\text{i}}b-b_{\text{i}}a\right)b}\exp\left(\frac{\mu a}{b}\right)E_{1}\left(\mu x_{0}+\frac{\mu a}{b}\right)\\ & \;+\frac{da_{\text{i}}-cb_{\text{i}}}{\left(a_{\text{i}}b-b_{\text{i}}a\right)b_{\text{i}}}\exp\left(\frac{\mu a_{\text{i}}}{b_{\text{i}}}\right)E_{1}\left(\mu x_{0}+\frac{\mu a_{\text{i}}}{b_{\text{i}}}\right) \end{array}$$

with *μ* > 0, $x_{0}\in R$ , and $a_{i}=I\left\{a\right\}$ , $b_{i}=I\left\{b\right\}$ , we can express Eq. (20) as

(31) $$\begin{array}{ll} \tau_{u\ell ,i}^{\text{b}} & =i\exp\left(D_{i}\right)G\left(s_{u\ell ,0},s_{u\ell }^{\prime }w_{i},\gamma_{i,0},\gamma_{i}^{\prime }w_{i},\mu_{i},x_{i}\right)\\ & \;-iG\left(s_{u\ell ,0},s_{u\ell }^{\prime }w_{i},\gamma_{i,0},\gamma_{i}^{\prime }w_{i},1,x_{i}\right), \end{array}$$

where $\mu_{u}=m_{u}^{*}/m_{\ell }^{*}$ , $\mu_{\ell }=m_{\ell }^{*}/m_{u}^{*}$ , $D_{u}=\;\hbar \delta_{u\ell ,k=0}/\left(k_{\text{B}}T_{u}\right)$ , $D_{\ell }=-\hbar \delta_{u\ell ,k=0}/\left(k_{\text{B}}T_{\ell }\right)$ , and $x_{i}=\max\left(0,D_{i}/\mu_{i}\right)$ . If we assume equal electron temperatures *T*_e_ in both subbands and neglect nonparabolicity as well as the energy dependence of dephasing (i.e., *μ*_*i*_ = 1, $s_{u\ell }^{\prime }=\gamma_{i}^{\prime }=0$ ), Eq. (30) simplifies to $G=c{\left(a_{i}a\right)}^{-1}\exp\left(-x_{0}\right)$ . Furthermore choosing the optical carrier frequency *ω*_c_ as the transition frequency *ω*_*uℓ*_, Eq. (31) becomes with *δ*_*uℓ*,*k*=0_ → 0

(32) $$\tau_{u\ell ,i}^{\text{b}}=\pm i\frac{\hbar }{k_{\text{B}}T_{\text{e}}}\frac{\gamma_{i,0}}{\gamma_{u\ell ,0}},$$

where the “+” and “−” sign is for *i* = *u* and *i* = *ℓ*, respectively. With Γ_*uℓ*_ = *γ*_*uℓ*_ and $\tau_{u\ell ,i}^{\text{np}}=\gamma_{u\ell }^{-1}$ , we recover the modified Maxwell–Bloch equations introduced in ref. [39] by assuming *γ*_*i*,0_ = *γ*_*uℓ*,0_/2.

### 3.3 Generalization to multiple levels

The procedure for deriving the effective parameters can straightforwardly be extended to optical and tunneling transitions involving multiple levels. This is the case if a laser transition has more than one upper or lower laser level, or for coherent coupling of the laser levels to other states by resonant tunneling. For the level scheme illustratively sketched in Figure 1, injection into the upper or extraction from the lower laser level may, e.g., be dominated by resonant tunneling, described in the model by a corresponding equation of the form Eq. (B.2). Given a subset of *N* levels in the quantum system which interact coherently, each corresponding off-diagonal DM element is governed by an evolution equation of the form

(33) $$\begin{array}{ll} \partial_{t}\sigma_{mn,k} & =-s_{mn,k}\sigma_{mn,k}+\underset{i\neq n}{\sum }\xi_{mn,in}\sigma_{mi,k}\\ & \;+\underset{i\neq m}{\sum }\xi_{mn,mi}\sigma_{in,k}. \end{array}$$

For a near-resonant optical transition between two levels *i* and *j*, *σ*_*ij*,**k**_ represents the corresponding off-diagonal DM element in RWA, i.e., *σ*_*ij*,**k**_ = *η*_*ij*,**k**_, and *s*_*ij*,**k**_ is given by Eq. (B.4). For a closely aligned pair of levels *i* and *j*, *σ*_*ij*,**k**_ = *ρ*_*ij*,**k**_, and *s*_*mn*,**k**_ = *γ*_*mn*,**k**_ + i*ω*_*mn*,**k**_. The constants *ξ*_*mn*,*ij*_ represent the coefficients in Eqs. (B.2) and (B.3), related to Ω_*ij*_ and *ɛ*_*ij*_, respectively.

For deriving the effective DM equations, the stationary carrier densities $\sigma_{ii,k}^{\text{o}}=\rho_{i,k}^{\text{o}}$ and the average optical intensity *I*_o_ at the operating point are extracted from the carrier transport simulations (if the operating point is close to threshold, an arbitrary small value for *I*_o_ can be assumed). The *ξ*_*mn*,*ij*_ related to the optical field are then obtained from

(34) $$\varepsilon_{ij}=d_{ij}{\left[2I_{\text{o}}/\left(\hbar^{2}\epsilon_{0}{cn}_{0}\right)\right]}^{1/2}$$

with the vacuum speed of light *c*, vacuum permittivity *ϵ*_0_ and refractive index *n*_0_. Writing down Eq. (33) for all non-zero off-diagonal DM elements *σ*_*mn*_ associated with the subset of coherently interacting levels and setting ∂_*t*_ = 0, a linear equation system is obtained which allows us to compute the stationary solutions $\sigma_{mn,k}^{\text{o}}$ . Similarly as in Section 3.2, both sides of Eq. (33) are divided by *s*_*mn*,**k**_, and subsequently, the **k** summation is performed. For a quantum well structure with in-plane isotropy, the **k** summation can be replaced by integration over the energy *w* defined in Eq. (28), see Section 3.2.3. Using Eq. (6), we define $\sigma_{ii}^{\text{o}}=\sum_{k}\rho_{i,k}^{\text{o}}$ and $\sigma_{mn}^{\text{o}}=\sum_{k}\sigma_{mn,k}^{\text{o}}$ . We introduce the effective parameters Γ_*mn*,*ij*_ via setting $\sum_{k}s_{mn,k}^{-1}\sigma_{ij,k}=\Gamma_{mn,ij}^{-1}\sigma_{ij}$ and using the stationary solutions for the *σ*_*ij*,**k**_. This yields as a generalization of Eq. (13)

(35) $$\Gamma_{mn,ij}=\sigma_{ij}^{\text{o}}/\underset{k}{\sum }s_{mn,k}^{-1}\sigma_{ij,k}^{\text{o}}.$$

Specifically, for **k** independent *s*_*mn*_, we obtain Γ_*mn*,*ij*_ = *s*_*mn*_. For more compact notation, we write Γ_*mn*,*mn*_ ≕ Γ_*mn*_. After multiplication with Γ_*mn*_, we obtain with $c_{mn,ij}=\Gamma_{mn}\Gamma_{mn,ij}^{-1}$ the effective DM equation

(36) $$\begin{array}{ll} \partial_{t}\sigma_{mn} & =-\Gamma_{mn}\sigma_{mn}+\underset{i\neq n}{\sum }\xi_{mn,in}c_{mn,mi}\sigma_{mi}\\ & \;+\underset{i\neq m}{\sum }\xi_{mn,mi}c_{mn,in}\sigma_{in}. \end{array}$$

Equation (36) contains the effect of nonparabolicity. For *σ*_*mn*_ describing optical transitions, Bloch gain may be included similarly as in Eq. (18) by defining

(37) $$c_{mn,ii}=\Gamma_{mn}\left(\Gamma_{mn,ii}^{-1}+\tau_{mn,i}^{\text{b}}\right)$$

with *i* = *m*, *n*, where $\tau_{mn,i}^{\text{b}}$ is given by Eq. (20). For the inclusion of SHB and coupling of the DM description to optical propagation equations, see Appendix C.

## 4 Examples

Nonparabolicity is usually much more pronounced in mid-infrared (MIR) than in terahertz QCLs, since the larger energy spacing between the upper and lower laser levels tends to enhance the difference between the effective masses. Furthermore, the nonparabolicity effect increases with electron temperature since higher **k** states get occupied. Thus, in the following we focus on high-temperature MIR QCL structures.

### 4.1 Analytical effective parameter model

**Figure 3:**
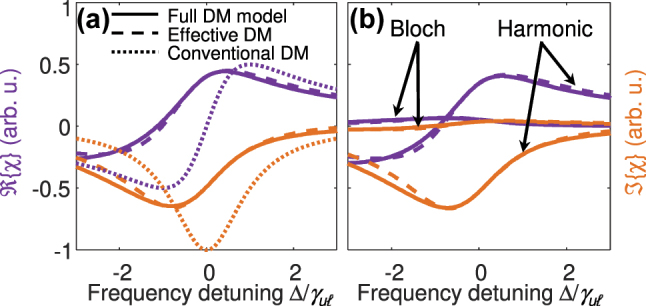
Calculated (a) susceptibility *χ* and (b) harmonic and Bloch contributions to *χ* as a function of the normalized frequency detuning Δ/*γ*_*uℓ*_ for a two-level system. The results from the analytical effective parameter model, introduced in Section 3.2.3, are compared to calculations based on the conventional discrete-level and the fully **k** resolved DM model.

In order to validate the analytical effective parameter model introduced in Section 3.2.3, we choose $m_{u}^{*}=1.2\;m_{\ell }^{*}=0.06\;m_{\text{e}}$ , *ρ*_*ℓ*_ = *ρ*_*u*_/3, *T*_*ℓ*_ = 1.5 *T*_*u*_ = 900 K, *γ*_*uℓ*_ = 10 ps^−1^ corresponding to a Lorentzian gain bandwidth of *γ*_*uℓ*_/*π* = 3.2 THz, and *γ*_*u*_ = *γ*_*ℓ*_ = *γ*_*uℓ*_/2. These are realistic values for MIR QCLs and give rise to a pronounced nonparabolicity. From Eqs. (18), (29) and (31), the effective parameter values $c_{u\ell ,u}^{\text{np}}=0.886+0.228i$ , $c_{u\ell ,\ell }^{\text{np}}=0.796+0.248i$ , $c_{u\ell ,u}^{\text{b}}=-0.013+0.054i$ , $c_{u\ell ,\ell }^{\text{b}}=0.012-0.035i$ , and Γ_*uℓ*_ = 14.2 − 5.2i ps^−1^ are obtained. For validating the effective model, we compare the frequency dependent susceptibility *χ* ∝ *η*_*uℓ*_/*ɛ*_*uℓ*_ computed from Eq. (21) with the result of the fully **k** dependent calculation, obtained by solving Eq. (11) in analogy to Eq. (21) and employing Eq. (6). In Figure 3(a), the obtained susceptibility is shown for the effective and fully **k** dependent model as well as for the conventional discrete-level DM equations, obtained by setting Γ_*uℓ*_ = *s*_*uℓ*_ and *c*_*uℓ*,*u*_ = *c*_*uℓ*,*ℓ*_ = 1 in Eq. (21). Figure 3(b) displays the harmonic and Bloch contributions to *χ*. Overall, we find good agreement between the full and the effective model. As expected, the conventional discrete-level DM equations do not capture the asymmetry and broadening of *χ* and *α* caused by nonparabolicity and the Bloch contribution.

### 4.2 Multilevel effective parameter model

**Figure 4:**
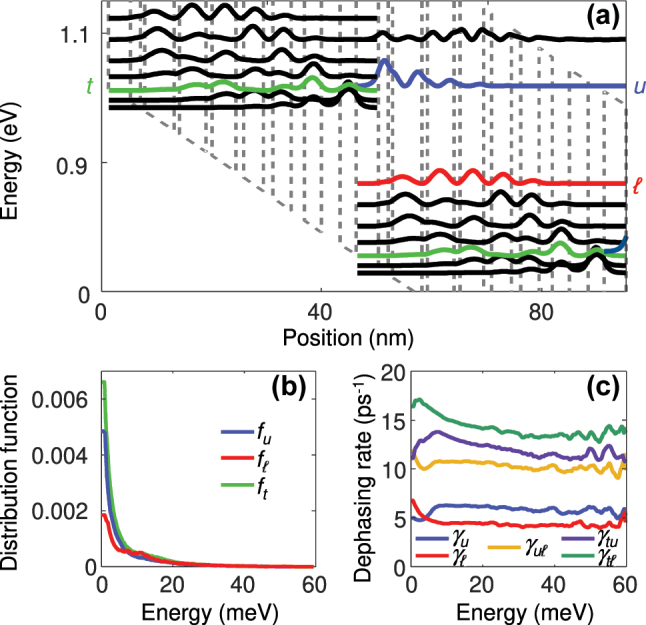
Carrier transport simulation results for the investigated QCL. (a) Conduction band profile with energy levels and probability densities. (b) Electron distribution functions $f_{i}\left(w\right)$ for the upper laser level (*u*), lower laser level (*ℓ*), and tunneling injector (*t*) as a function of the energy *w*, defined in Eq. (28). (c) Dephasing rates $\gamma_{i}\left(w\right)$ and $\gamma_{ij}\left(w\right)$ .

As a test case for the general effective multilevel DM model of Section 3.3, we choose a diagonal bound-to-continuum room temperature QCL design emitting at 8.5 μm [66], which has been widely used as a reference structure for validating modeling approaches [65], [66], [67]. In Figure 4(a), the energy levels of a representative stage, which have been computed with a Schrödinger–Poisson solver, are displayed. Furthermore, DM-Monte Carlo carrier transport simulations have been performed [56], [65]. The simulated energy dependent distribution functions and dephasing rates are shown in Figure 4(b) and (c), respectively. The electron dispersion relation is here modeled using Eq. (1). The effective masses of the upper and lower laser levels *u* and *ℓ* are 0.0604 and 0.0547, giving rise to a pronounced nonparabolicity. Additionally, Bloch gain between the laser levels and the coherent coupling of the upper laser level to the tunneling injector *t* contribute to the asymmetry. For this subset of coupled subbands, Eq. (33) becomes

(38) $$\begin{array}{ll} \partial_{t}\left(\begin{array}{c} \eta_{u\ell ,k}\\ \eta_{t\ell ,k}\\ \rho_{tu,k} \end{array}\right) & =-\left(\begin{array}{ccc} s_{u\ell ,k} & i\Omega_{tu} & 0\\ i\Omega_{tu} & s_{t\ell ,k} & \frac{i}{2}\varepsilon_{u\ell }\\ 0 & \frac{i}{2}\varepsilon_{u\ell }^{*} & s_{tu,k} \end{array}\right)\left(\begin{array}{c} \eta_{u\ell ,k}\\ \eta_{t\ell ,k}\\ \rho_{tu,k} \end{array}\right)\\ & \;+\left(\begin{array}{c} \frac{i}{2}\varepsilon_{u\ell }\left(\rho_{\ell ,k}-\rho_{u,k}\right)\\ 0\\ i\Omega_{tu}\left(\rho_{t,k}-\rho_{u,k}\right) \end{array}\right), \end{array}$$

with *ρ*_*i*,**k**_ = *Sf*_*i*,**k**_ where the scaling factor *S* introduced in Eq. (2) can be freely chosen. Taking advantage of the in-plane isotropy, we represent the **k** dependence in terms of the energy variable *w* introduced in Eq. (28). The $\rho_{i}\left(w\right)$ and $s_{ij}\left(w\right)$ are provided by the carrier transport simulations at the operating point as shown in Figure 4; furthermore, *ℏ*Ω_*tu*_ = 3.2 meV is obtained. Assuming operation close to threshold, an arbitrary small value for *ɛ*_*uℓ*_ can be assumed. Setting ∂_*t*_ = 0, a linear equation system is obtained for $\sigma_{ij}^{\text{o}}\left(w\right)=\eta_{u\ell }\left(w\right)$ , $\eta_{t\ell }\left(w\right)$ and $\rho_{tu}\left(w\right)$ . Plugging the results in Eq. (35) and replacing the summation over **k** by integration over *w*, the correction coefficients Γ_*mn*,*ij*_ are obtained. The reduced effective DM equations are then given by

(39) $$\begin{array}{ll} \partial_{t}\left(\begin{array}{c} \eta_{u\ell }\\ \eta_{t\ell }\\ \rho_{tu} \end{array}\right) & =-\left(\begin{array}{ccc} \Gamma_{u\ell } & ic_{u\ell ,t\ell }\Omega_{tu} & 0\\ ic_{t\ell ,u\ell }\Omega_{tu} & \Gamma_{t\ell } & \frac{i}{2}c_{t\ell ,tu}\varepsilon_{u\ell }\\ 0 & \frac{i}{2}c_{tu,t\ell }\varepsilon_{u\ell }^{*} & \Gamma_{tu} \end{array}\right)\\ & \;\cdot \left(\begin{array}{c} \eta_{u\ell }\\ \eta_{t\ell }\\ \rho_{tu} \end{array}\right)+\left(\begin{array}{c} \frac{i}{2}\varepsilon_{u\ell }\left(c_{u\ell ,\ell \ell }\rho_{\ell }-c_{u\ell ,uu}\rho_{u}\right)\\ 0\\ i\Omega_{tu}\left(c_{tu,tt}\rho_{t}-c_{tu,uu}\rho_{u}\right) \end{array}\right), \end{array}$$

where $c_{mn,ij}=\Gamma_{mn}\Gamma_{mn,ij}^{-1}$ , and Eq. (37) has been used to include Bloch gain.

**Figure 5:**
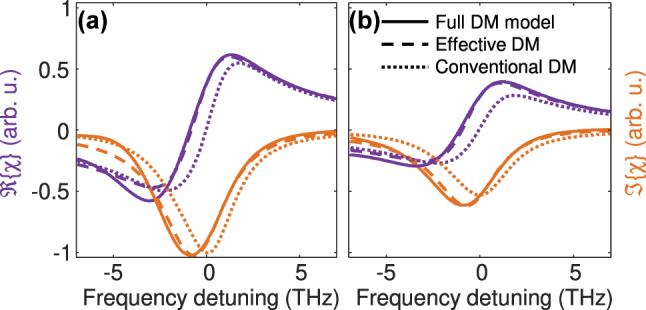
Susceptibility *χ* for a three-level system, consisting of the laser levels and a tunneling injector, as a function of the frequency detuning $\Delta /\left(2\pi \right)$ for the (a) unsaturated and (b) saturated case. The results from the generalized effective parameter model, introduced in Section 3.3, are compared to calculations based on the conventional discrete-level and the fully **k** resolved DM model.

For validating Eq. (39), we again compare the frequency dependent susceptibility *χ* ∝ *η*_*uℓ*_/*ɛ*_*uℓ*_. Similarly as for Eq. (21), the frequency dependent *η*_*uℓ*_ is obtained by inserting a frequency-shifted field $\varepsilon_{u\ell }\to \varepsilon_{u\ell }\left(\Delta \right)\exp\left(-i\Delta t\right)$ and corresponding optical DM elements $\eta_{ij}\to \eta_{ij}\left(\Delta \right)\exp\left(-i\Delta t\right)$ into Eq. (39), where Δ = *ω* − *ω*_c_. Cancelling the factor $\exp\left(-i\Delta t\right)$ and setting ∂_*t*_ = 0 yields a linear equation system for the stationary solution, which is solved in dependence of Δ to obtain $\eta_{u\ell }\left(\Delta \right)$ . The exact result is obtained by computing $\eta_{u\ell ,k}\left(\Delta \right)$ in an analogous manner from Eq. (38), and performing the **k** summation according to Eq. (6). As can be seen from Figure 5(a), the susceptibility obtained with the effective discrete-level DM model agrees well with exact result of the fully **k** dependent DM simulation, while the conventional discrete-level DM equations, obtained by setting Γ_*mn*_ = *s*_*mn*_ and *c*_*mn*,*ij*_ = 1 in Eq. (39), do not provide a good fit. The results shown in Figure 5(b) are for the same device, but the carrier transport simulations have been performed under lasing conditions [65], resulting in gain saturation. Again, the effective DM model yields good agreement with the exact results.

### 4.3 Dynamic simulations

**Figure 6:**
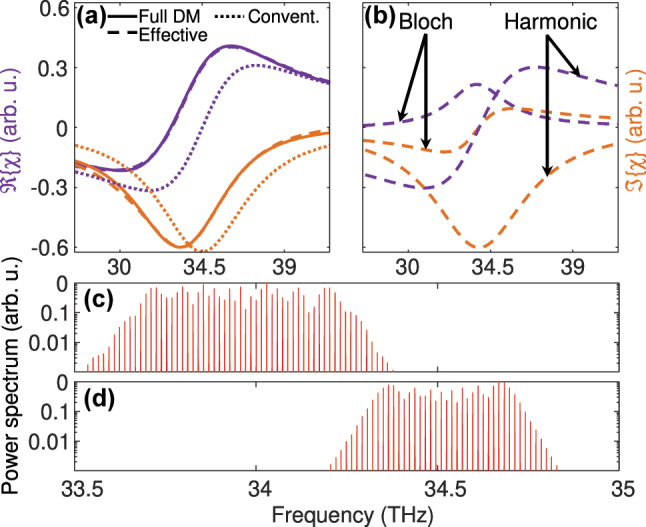
Simulation results for QCL multimode operation: (a) active region susceptibility *χ* at threshold, calculated with different models; (b) Bloch and harmonic contributions to *χ* according to the effective parameter model; (c) and (d) multimode spectra obtained with the (c) effective and (d) conventional Maxwell-DM approach.

Finally, we present simulations based on the effective Maxwell-DM approach to assess the numerical performance of the model, and to investigate the influence of nonparabolicity and Bloch gain on the QCL dynamics. For the dynamic simulations, Eqs. (C.3)–(C.9) in Appendix C are solved on a spatiotemporal grid, using an explicit 3rd order Adams-Bashforth method for Eqs. (C.3)–(C.7) and a finite difference scheme for Eq. (C.8) [25]. To obtain realistic results, SHB and group velocity dispersion are included in the model. Furthermore, spontaneous emission noise is considered in Eq. (C.8) to account for the associated field fluctuations and to emulate the buildup of lasing. As an exemplary structure, we choose a Fabry–Perot cavity with a vertical two-phonon resonance active region, featuring room temperature operation at around 9 μm [68], [69]. This design has for example been used for investigating the formation of dense and harmonic multimode spectra under different driving conditions [70]. Similarly as in Figure 4(a), we model injection into the upper laser level by tunneling though the thick injection barrier. Thus, the coherent coupling between the injection, upper and lower laser levels can again be described by Eq. (39). As outlined in Section 4.2, the Hamiltonian matrix elements, scattering/dephasing rates and effective parameters are extracted from carrier transport simulations. In Figure 6(a), the computed susceptibility at lasing threshold is shown as a function of frequency for the same models as in Figure 5, again yielding excellent agreement between the effective and full DM approach. In addition, the Bloch and harmonic contributions are displayed in Figure 6(b) for the effective DM model. Both the nonparabolicity and the Bloch gain contribute to the gain asymmetry, resulting in a noticeable shift of the gain peak to lower frequencies.

In the following, we focus on dense multimode operation, since the emergence of harmonic spectra in free-running lasers is quite elusive, critically depending on the drive history, sample used and other factors [19], [49], [70]. Exemplarily, we investigate the effect of nonparabolicity, since the influence of Bloch gain on the QCL dynamics has already been studied in detail for a similar active region design [39]. In Figure 6(c) and (d), simulation results of the effective and conventional Maxwell-DM model are shown for a moderate two-facet output power of ∼50mW . Both approaches yield a dense multimode spectrum already slightly above threshold, as also observed in experiment [70]. Although multimode operation in Fabry–Perot cavities is largely governed by SHB [71], [72], the spectrum is clearly broader for the effective model, featuring a 20-dB bandwidth of 0.71 THz (i.e., 2.1 % relative bandwidth) versus 0.54 THz (1.6 %) for the conventional approach. This illustrates the contribution of nonparabolicity-induced linewidth enhancement to multimode formation. In addition, the spectrum obtained with the effective model is downshifted in frequency and thus agrees somewhat better with the experimentally observed wavelength range [70], which however has been found to depend significantly on the growth process and facility [73]. Simulations at higher output powers likewise yield broader and frequency-downshifted spectra for the effective model, providing a better overall match to experiment as expected. The numerical stability and efficiency of the effective Maxwell-DM approach has been further validated by applying it to other test structures. Since the effective parameters can directly be extracted from the carrier transport simulations and the effective Maxwell-DM equations have the same complexity as the conventional model, the computational cost is comparable for both approaches. Thus, the effective Maxwell-DM model is well-suited for dynamic QCL modeling, and specifically for the investigation of operating regimes where gain asymmetry plays a pronounced role, such as comb and soliton formation in ring cavities [21], [22], [23], [24], [39], [43], [45], [74] and harmonic operation [15], [16], [17], [18], [19], [20], [70].

## 5 Conclusions

An effective DM model has been derived for unipolar quantum optoelectronic devices by adequate summation over the electron wavevector, which characterizes the free carrier motion in the directions without quantum confinement. The resulting effective discrete-level DM equations differ from models for true discrete-level quantum systems, such as quantum dots, by containing additional effective parameters. This extended description includes gain asymmetry and linewidth enhancement by considering effects such as nonparabolicity and Bloch gain. Here, the effective parameters are extracted from carrier transport simulations, providing a self-consistent model without phenomenological parameters. Good agreement with fully wavevector dependent simulations is found. By coupling the DM description to optical propagation equations, an effective Maxwell-DM model is obtained for the combined optical and electronic device dynamics. The approach is validated by exemplary QCL simulations, achieving numerical performance comparable to the conventional discrete-level model while offering greatly improved accuracy and versatility. Thus, the effective Maxwell-DM equations are well-suited for the theoretical investigation of dynamic operating regimes, such as comb generation in ring cavities or the formation of solitons and harmonic states. The predictive power of the model may be further enhanced by taking into account the contributions of non-resonant optical transitions to linewidth enhancement. Perspectively, an adaption of the presented approach to bipolar quantum optoelectronic devices would be highly attractive. In this context, interband cascade lasers (ICLs) [75] are of particular interest, since they have recently shown great potential for the generation of dynamic waveforms in the mid-infrared regime, such as short pulses [76], broadband frequency combs [77], [78] and harmonic comb states [79]. Suitable approaches for microscopic carrier transport simulations, required as input for the self-consistent dynamic device model introduced in this paper, are meanwhile available for ICLs [80]. Generally, for bipolar optoelectronic devices a main challenge is that computing the effective parameter integrals may involve divergence problems [32], which must be adequately handled.

## Appendix A: Collision term model

The collision terms ${\left[\partial_{t}\rho_{mn,k}\right]}_{\text{col}}$ and ${\left[\partial_{t}\rho_{n,k}\right]}_{\text{col}}$ in Eq. (4), which account for dissipative processes, can be implemented based on a full quantum description as is done in quantum-kinetic approaches [81], [82], [83], [84], or under certain assumptions by employing a more amenable relaxation rate model [25], [81], [85]. Using the latter approach, we use a dissipation model of the form [25], [52]

(A.1a)
$${\left[\partial_{t}\rho_{mn,k}\right]}_{\text{col}}=-\gamma_{mn,k}\rho_{mn,k},$$

(A.1b)
$$\begin{array}{ll} {\left[\partial_{t}\rho_{n,k}\right]}_{\text{col}} & =\underset{i}{\sum }\underset{q}{\sum }\left[W_{iq,nk}\rho_{i,q}\left(1-f_{n,k}\right)\right.\\ & \left.\;-W_{nk,iq}\rho_{n,k}\left(1-f_{i,q}\right)\right], \end{array}$$

where the terms $\left(1-f_{n,k}\right)$ and $\left(1-f_{i,q}\right)$ account for Pauli blocking. The dephasing rate is denoted by *γ*_*mn*,**k**_, with *γ*_*mn*,**k**_ = *γ*_*nm*,**k**_. The scattering rates *W*_*n* **k**,*i* **q**_ include all the relevant scattering mechanisms, i.e., $W_{nk,iq}=\sum_{s}W_{nk,iq}^{\left(s\right)}$ where the index *s* labels the different mechanisms. The $W_{nk,iq}^{\left(s\right)}$ can for example be derived from microscopic models, and may themselves depend on the carrier distribution, e.g., for carrier-carrier scattering [52].

## Appendix B: Rotating wave approximation

The evolution equations for the DM elements in RWA are obtained from Eq. (4) by making the substitutions given in Eq. (5) and discarding the rapidly oscillating terms. For the diagonal DM elements, we obtain

(B.1)
$$\begin{array}{ll} \partial_{t}\rho_{nn,k} & =i\underset{i\neq n}{\sum }\left(\Omega_{in}\rho_{ni,k}-\Omega_{ni}\rho_{in,k}\right)\\ & \;+\underset{\begin{array}{c} i\\ \omega_{ni,k}>0 \end{array}}{\sum }I\left\{\varepsilon_{ni}^{*}\eta_{ni,k}\right\}+\underset{\begin{array}{c} i\\ \omega_{ni,k}<0 \end{array}}{\sum }I\left\{\varepsilon_{ni}\eta_{ni,k}\right\}\\ & \;+{\left[\partial_{t}\rho_{nn,k}\right]}_{\text{col}}. \end{array}$$

The off-diagonal DM elements for transitions between closely aligned levels $\left(\left|\omega_{mn,k}\right|\ll \omega_{\text{c}}\right)$ are with Eq. (A.1a) obtained as

(B.2)
$$\begin{array}{ll} \partial_{t}\rho_{mn,k} & =-\left(\gamma_{mn,k}+i\omega_{mn,k}\right)\rho_{mn,k}\\ & \;+i\underset{i\neq n}{\sum }\Omega_{in}\rho_{mi,k}-i\underset{i\neq m}{\sum }\Omega_{mi}\rho_{in,k}\\ & \;+\frac{i}{2}\underset{\begin{array}{c} i\\ \omega_{in,k}>0 \end{array}}{\sum }\varepsilon_{mi}^{*}\eta_{in,k}+\frac{i}{2}\underset{\begin{array}{c} i\\ \omega_{in,k}<0 \end{array}}{\sum }\varepsilon_{mi}\eta_{in,k}\\ & \;-\frac{i}{2}\underset{\begin{array}{c} i\\ \omega_{mi,k}>0 \end{array}}{\sum }\varepsilon_{in}^{*}\eta_{mi,k}-\frac{i}{2}\underset{\begin{array}{c} i\\ \omega_{mi,k}<0 \end{array}}{\sum }\varepsilon_{in}\eta_{mi,k}. \end{array}$$

For the off-diagonal DM elements in near-resonance with the optical field (*ω*_*mn*,**k**_ ≈ *ω*_c_) with *ω*_*mn*,**k**_ > 0, we obtain with Eqs. (4) and (A.1a) in the RWA

(B.3)
$$\begin{array}{ll} \partial_{t}\eta_{mn,k} & =-s_{mn,k}\eta_{mn,k}\\ & \;+i\underset{\begin{array}{c} i\neq n\\ \omega_{mi,k}>0 \end{array}}{\sum }\Omega_{in}\eta_{mi,k}-i\underset{\begin{array}{c} i\neq m\\ \omega_{in,k}>0 \end{array}}{\sum }\Omega_{mi}\eta_{in,k}\\ & \;+\frac{i}{2}\underset{i}{\sum }\varepsilon_{mi}\rho_{in,k}-\frac{i}{2}\underset{i}{\sum }\varepsilon_{in}\rho_{mi,k}, \end{array}$$

with

(B.4)
$$s_{mn,k}=\gamma_{mn,k}+i\left(\omega_{mn,k}-\omega_{\text{c}}\right)=\gamma_{mn,k}+i\delta_{mn,k}.$$

The remaining elements with *ω*_*mn*,**k**_ < 0 are then obtained using $\eta_{mn,k}=\eta_{nm,k}^{*}$ . Importantly, for compatibility with the RWA, we assume that in Eqs. (B.1)–(B.3) all off-diagonal DM elements *ρ*_*ij*,**k**_ refer to closely aligned levels ( $\left|\omega_{ij,k}\right|\ll \omega_{\text{c}}$ ), and all *η*_*ij*,**k**_ are associated with near-resonant optical transitions $\left|\omega_{ij,k}\right|\approx \omega_{\text{c}}$ . The remaining off-diagonal DM elements are set to 0 in our RWA model, and transitions between the corresponding levels are assumed to be exclusively mediated by incoherent scattering, considered in Eq. (B.1) by the collision term.

## Appendix C: Optical propagation and spatial hole burning

For realistic device simulations, SHB in form of an inversion grating, resulting from the interference of counterpropagating waves in a resonator, must be considered [25], [60], [61], [71], [86]. In the following, we proceed as in ref. [47]. We note that the DM elements are regarded as position dependent, i.e., $\rho_{mn}\;\left(x,t\right)$ and $\eta_{u\ell }\;\left(x,t\right)$ denote the DM elements of a representative quantum system at position *x* in the resonator. Since SHB is counteracted by diffusion, we add a term $\partial_{t}\rho_{mn}=\ldots +D_{mn}\partial_{x}^{2}\rho_{mn}$ to the evolution equation of a given DM element *ρ*_*mn*_ (and analogously for *η*_*mn*_), where *D*_*mn*_ denotes the corresponding diffusion coefficient. Including the grating to lowest order, we write the spatial dependence of the optical field as $\varepsilon_{mn}=\varepsilon_{mn}^{+}\exp\left(i\beta x\right)+\varepsilon_{mn}^{-}\exp\left(-i\beta x\right)$ , where *β* is the propagation constant of the guided mode and the envelopes $\varepsilon_{mn}^{\pm }\left(x,t\right)$ are assumed to vary slowly in space and time. For introducing $\eta_{mn}^{\pm }\left(x,t\right)$ , we proceed analogously. The remaining DM elements are represented as $\rho_{mn}=\rho_{mn}^{0}+\sum_{\pm }\rho_{mn}^{2\pm }\exp\left(\pm 2i\beta x\right)$ . For *m* = *n*, we have $\rho_{n}^{2+}={\left(\rho_{n}^{2-}\right)}^{*}$ (with $\rho_{n}^{2\pm }≔\rho_{nn}^{2\pm }$ ).

### C.1 Two-level model

Discarding higher order oscillation terms, we obtain from Eqs. (10) and (18)

(C.1)
$$\begin{array}{ll} \partial_{t}\rho_{n}^{0} & =\mathrm{sgn}\left(\omega_{nm}\right)I\left\{{\left(\varepsilon_{u\ell }^{+}\right)}^{*}\eta_{u\ell }^{+}+{\left(\varepsilon_{u\ell }^{-}\right)}^{*}\eta_{u\ell }^{-}\right\}\\ & \;+\underset{i\neq n}{\sum }r_{in}\rho_{i}^{0}-\rho_{n}^{0}\underset{i\neq n}{\sum }r_{ni},\\ \partial_{t}\rho_{n}^{2\pm } & =\frac{i}{2}\mathrm{sgn}\left(\omega_{nm}\right)\left[\varepsilon_{u\ell }^{\pm }{\left(\eta_{u\ell }^{\mp }\right)}^{*}-{\left(\varepsilon_{u\ell }^{\mp }\right)}^{*}\eta_{u\ell }^{\pm }\right]\\ & \;+\underset{i\neq n}{\sum }r_{in}\rho_{i}^{2\pm }-\rho_{n}^{2\pm }\underset{i\neq n}{\sum }r_{ni}-4\beta^{2}D_{nn}\rho_{n}^{2\pm }, \end{array}$$

with *n* = *u*, *m* = *ℓ* and *m* = *u*, *n* = *ℓ*, respectively, and

(C.2)
$$\begin{array}{ll} \partial_{t}\eta_{u\ell }^{\pm } & =-\Gamma_{u\ell }\eta_{u\ell }^{\pm }+\frac{i}{2}\varepsilon_{u\ell }^{\pm }\left(c_{u\ell ,\ell }^{\text{np}}\rho_{\ell }^{0}-c_{u\ell ,u}^{\text{np}}\rho_{u}^{0}\right)\\ & \;+\frac{i}{2}\varepsilon_{u\ell }^{\mp }\left(c_{u\ell ,\ell }\rho_{\ell }^{2\pm }-c_{u\ell ,u}\rho_{u}^{2\pm }\right)-\beta^{2}D_{u\ell }\eta_{u\ell }^{\pm }. \end{array}$$

We note that in this model, similarly as in previous work [39], [44], a possible influence of SHB on the parameters Γ_*uℓ*_, *c*_*uℓ*,*u*_ and *c*_*uℓ*,*ℓ*_ has been neglected.

### C.2 Generalized multilevel model

Analogously, SHB can be included into the generalized multilevel model. From Eq. (7), we obtain for the occupations

(C.3)
$$\begin{array}{ll} \partial_{t}\rho_{nn}^{0} & =2\underset{i\neq n}{\sum }I\left\{\Omega_{ni}\rho_{in}^{0}\right\}\\ & \;-\underset{\begin{array}{c} i\\ \omega_{ni}>0 \end{array}}{\sum }I\left\{\eta_{in}^{-}\varepsilon_{ni}^{+}+\eta_{in}^{+}\varepsilon_{ni}^{-}\right\}\\ & \;+\underset{\begin{array}{c} i\\ \omega_{ni}<0 \end{array}}{\sum }I\left\{\eta_{ni}^{+}\varepsilon_{ni}^{-}+\eta_{ni}^{-}\varepsilon_{ni}^{+}\right\}\\ & \;+\underset{i\neq n}{\sum }r_{in}\rho_{ii}^{0}-\rho_{nn}^{0}\underset{i\neq n}{\sum }r_{ni}, \end{array}$$

(C.4)
$$\begin{array}{ll} \partial_{t}\rho_{nn}^{2\pm } & =i\underset{i\neq n}{\sum }\left(\Omega_{in}\rho_{ni}^{2\pm }-\Omega_{ni}\rho_{in}^{2\pm }\right)\\ & \;+\frac{i}{2}\underset{\begin{array}{c} i\\ \omega_{ni}>0 \end{array}}{\sum }\left[\varepsilon_{ni}^{\pm }\eta_{in}^{\pm }-{\left(\varepsilon_{ni}^{\mp }\right)}^{*}\eta_{ni}^{\pm }\right]\\ & \;+\frac{i}{2}\underset{\begin{array}{c} i\\ \omega_{ni}<0 \end{array}}{\sum }\left[{\left(\varepsilon_{ni}^{\mp }\right)}^{*}\eta_{in}^{\pm }-\varepsilon_{ni}^{\pm }\eta_{ni}^{\pm }\right]\\ & \;+\underset{i\neq n}{\sum }r_{in}\rho_{ii}^{2\pm }-\rho_{nn}^{2\pm }\underset{i\neq n}{\sum }r_{ni}-4\beta^{2}D_{nn}\rho_{nn}^{2\pm }, \end{array}$$

with $\rho_{nn}^{2+}={\left(\rho_{nn}^{2-}\right)}^{*}$ . The off-diagonal DM elements for transitions between closely aligned levels ( $\left|\omega_{mn,k}\right|\ll \omega_{\text{c}}$ ) are described by

(C.5)
$$\begin{array}{ll} \partial_{t}\rho_{mn}^{0} & =-\Gamma_{mn}\rho_{mn}^{0}\\ & \;+i\underset{i\neq n}{\sum }\Omega_{in}c_{mn,mi}\rho_{mi}^{0}-i\underset{i\neq m}{\sum }\Omega_{mi}c_{mn,in}\rho_{in}^{0}\\ & \;+\frac{i}{2}\underset{\begin{array}{c} i\\ \omega_{in}>0 \end{array}}{\sum }c_{mn,in}\left[{\left(\varepsilon_{mi}^{+}\right)}^{*}\eta_{in}^{+}+{\left(\varepsilon_{mi}^{-}\right)}^{*}\eta_{in}^{-}\right]\\ & \;+\frac{i}{2}\underset{\begin{array}{c} i\\ \omega_{in}<0 \end{array}}{\sum }c_{mn,in}\left(\varepsilon_{mi}^{-}\eta_{in}^{+}+\varepsilon_{mi}^{+}\eta_{in}^{-}\right)\\ & \;-\frac{i}{2}\underset{\begin{array}{c} i\\ \omega_{mi}>0 \end{array}}{\sum }c_{mn,mi}\left[{\left(\varepsilon_{in}^{+}\right)}^{*}\eta_{mi}^{+}+{\left(\varepsilon_{in}^{-}\right)}^{*}\eta_{mi}^{-}\right]\\ & \;-\frac{i}{2}\underset{\begin{array}{c} i\\ \omega_{mi}<0 \end{array}}{\sum }c_{mn,mi}\left(\varepsilon_{in}^{-}\eta_{mi}^{+}+\varepsilon_{in}^{+}\eta_{mi}^{-}\right), \end{array}$$

(C.6)
$$\begin{array}{ll} \partial_{t}\rho_{mn}^{2\pm } & =-\left(\Gamma_{mn}+4\beta^{2}D_{mn}\right)\rho_{mn}^{2\pm }\\ & \;+i\underset{i\neq n}{\sum }\Omega_{in}c_{mn,mi}\rho_{mi}^{2\pm }-i\underset{i\neq m}{\sum }\Omega_{mi}c_{mn,in}\rho_{in}^{2\pm }\\ & \;+\frac{i}{2}\underset{\begin{array}{c} i\\ \omega_{in}>0 \end{array}}{\sum }{\left(\varepsilon_{mi}^{\mp }\right)}^{*}c_{mn,in}\eta_{in}^{\pm }+\frac{i}{2}\underset{\begin{array}{c} i\\ \omega_{in}<0 \end{array}}{\sum }\varepsilon_{mi}^{\pm }c_{mn,in}\eta_{in}^{\pm }\\ & \;-\frac{i}{2}\underset{\begin{array}{c} i\\ \omega_{mi}>0 \end{array}}{\sum }{\left(\varepsilon_{in}^{\mp }\right)}^{*}c_{mn,mi}\eta_{mi}^{\pm }-\frac{i}{2}\underset{\begin{array}{c} i\\ \omega_{mi}<0 \end{array}}{\sum }\varepsilon_{in}^{\pm }c_{mn,mi}\eta_{mi}^{\pm }, \end{array}$$

thus fulfilling $\rho_{mn}^{0}={\left(\rho_{nm}^{0}\right)}^{*}$ , $\rho_{mn}^{2+}={\left(\rho_{nm}^{2-}\right)}^{*}$ . Regarding the off-diagonal DM elements in near-resonance with the optical field (*ω*_*mn*_ ≈ *ω*_c_), we obtain for *ω*_*mn*_ > 0

(C.7)
$$\begin{array}{ll} \partial_{t}\eta_{mn}^{\pm } & =-\left(\Gamma_{mn}+\beta^{2}D_{mn}\right)\eta_{mn}^{\pm }\\ & \;+i\underset{\begin{array}{c} i\neq n\\ \omega_{mi}>0 \end{array}}{\sum }\Omega_{in}c_{mn,mi}\eta_{mi}^{\pm }-i\underset{\begin{array}{c} i\neq m\\ \omega_{in}>0 \end{array}}{\sum }\Omega_{mi}c_{mn,in}\eta_{in}^{\pm }\\ & \;+\frac{i}{2}\underset{i}{\sum }c_{mn,in}\left(\varepsilon_{mi}^{\pm }\rho_{in}^{0}+\varepsilon_{mi}^{\mp }\rho_{in}^{2\pm }\right)\\ & \;-\frac{i}{2}\underset{i}{\sum }c_{mn,mi}\left(\varepsilon_{in}^{\pm }\rho_{mi}^{0}+\varepsilon_{in}^{\mp }\rho_{mi}^{2\pm }\right), \end{array}$$

and use $\eta_{nm}^{\pm }={\left(\eta_{mn}^{\mp }\right)}^{*}$ to compute the remaining DM elements.

For the simulation of devices featuring a modulated bias $u\left(x,t\right)$ along the waveguide, as applies, e.g., for actively mode-locked QCLs, the model has to be somewhat generalized by treating Ω_*mn*_(*u*) and *ω*_*mn*_(*u*) as *u* dependent parameters [47]. This description can also be extended to the scattering and dephasing rates *r*_*mn*_(*u*) and *γ*_*mn*_(*u*), assuming that they follow the bias change instantaneously. With *s*_*mn*_ = *γ*_*mn*_ + i*ω*_*mn*_ for closely aligned levels (Eqs. (C.5), (C.6)) and $s_{mn}=\gamma_{mn}+i\left(\omega_{mn}-\omega_{\text{c}}\right)$ for near-resonant optical transitions (Eq. (C.7)), we can then approximately write $\Gamma_{mn}\left(u\right)=s_{mn}\left(u\right)\Gamma_{mn}^{\text{o}}/s_{mn}^{\text{o}}$ , where the superscript o indicates the values at the operating point. For small modulations, a first order Taylor expansion of the bias dependent parameters around the DC value of *u* is sufficient [47]. As described in Section 3, the *c*_*mn*,*ij*_ are evaluated at the operating point and are thus modulation independent.

### C.3 Coupling to optical propagation equation

The Maxwell-DM equations, forming a closed model for the combined optical and electronic dynamics in the device, are obtained by coupling Eqs. (C.1) and (C.2) or (C.3)–(C.7) to the optical propagation equation [25]

(C.8)
$$v_{\text{g}}^{-1}\partial_{t}E^{\pm }\pm \partial_{x}E^{\pm }=-\frac{i}{2}\beta_{2}\partial_{t}^{2}E^{\pm }-\frac{a}{2}E^{\pm }+ip_{\pm }+S_{\pm }^{\text{sp}}.$$

Here, *a*, *v*_g_ and *β*_2_ are the waveguide power loss coefficient, group velocity, and background group velocity dispersion coefficient, and $\varepsilon_{u\ell }^{\pm }=\hbar^{-1}d_{u\ell }E^{\pm }$ . If the DM is normalized such that ∑_*n*_ *ρ*_*n*_ = 1 for each representative quantum system, the polarization term *p*_±_ in Eq. (C.8) is given by

(C.9)
$$p_{\pm }=\frac{n_{3\text{D}}\omega_{\text{c}}^{2}}{\epsilon_{0}\beta c^{2}}\Gamma \underset{\omega_{mn}>0}{\sum }d_{nm}\eta_{mn}^{\pm },$$

with the electron number density *n*_3D_, vacuum speed of light *c*, vacuum permittivity *ϵ*_0_, and overlap factor Γ. For a single optical transition, we have *m* = *u*, *n* = *ℓ*. $S_{\pm }^{\text{sp}}$ represents spontaneous emission, which is numerically modeled as distributed random noise [87].
